# Therapeutic benefits of Indole-3-Carbinol in adjuvant-induced arthritis and its protective effect against methotrexate induced-hepatic toxicity

**DOI:** 10.1186/s12906-018-2408-1

**Published:** 2018-12-19

**Authors:** Hiba Hasan, Hanan Ismail, Youmna El-Orfali, Ghada Khawaja

**Affiliations:** Department of Biological Sciences, Faculty of Science, Beirut Arab University, Debbieh, Lebanon

**Keywords:** Indole-3-carbinol, Adjuvant-induced arthritis, Anti-inflammatory, Anti-arthritic, Anti-oxidant, Hepatoprotective

## Abstract

**Background:**

Rheumatoid arthritis (RA) being an incapacitating disease requires early effective intervention. Considering Methotrexate (MTX)- the first line of treatment for RA- intoxicates the liver; therefore, alternative therapies with similar efficacy yet lower cytotoxicity are desired. Indole-3-Carbinol (I3C) which is found in cruciferous vegetables was examined for its possible therapeutic potentials in comparison with MTX by investigating its anti-inflammatory, anti-arthritic, anti-oxidant, and hepatoprotective potentials in adjuvant-induced arthritis (AIA) rat model.

**Methods:**

Arthritis was induced in Sprague Dawley rats by injection of Complete Freund’s Adjuvant (CFA). Arthritic rats were treated with I3C and/or MTX. To examine the anti-inflammatory and anti-arthritic effect, the following parameters were assessed: body weight, macroscopic scoring of the hind paw, the level of the pivotal cytokines (TNF-α, IL-6) heavily involved in the pathogenesis, spleen index, and erythrocyte sedimentation rate. At a histological level, the tibiotarsal joint was stained with several specific stains. To assess the hepatoprotective and anti-oxidant effects, several oxidative stress parameters were monitored, and the liver histology was examined.

**Results:**

Both I3C and MTX attenuated the inflammation that was aggravated by arthritis by downregulating the inflammatory markers and mediators and alleviating the histopathological changes affecting the tibiotarsal joint. I3C attenuated the liver impairment that was initiated by arthritis and MTX treatment. It did so by downregulating the pro-oxidants and up-regulating the anti-oxidant defenses and by reducing the pathological changes affecting the liver.

**Conclusion:**

Our results suggest that I3C is as potent as MTX as an anti-inflammatory and anti-arthritic agent. In addition, I3C does so while protecting the liver from damage as opposed to MTX.

## Background

Rheumatoid Arthritis (RA) is a progressive, chronic, autoimmune disease that is associated with inflammation affecting the lining of the joints, articular cartilage and bones. [1]. Infiltration of several mononuclear cells results in the release of an array of pro-inflammatory cytokines (TNF-α, IL-6 and IL-1β). The imbalance between pro and anti-inflammatory cytokines in RA eventually causes the synovial membrane to form a thickened area called pannus that has the ability to invade nearby cartilage and bone [2]. Moreover, the excessive release of cytokines can lead to systemic inflammation which can lead to deleterious consequences on several organs including the liver [3]. The overproduction of pro-inflammatory cytokines stimulates neutrophils and activates macrophages to secrete reactive oxygen species (ROS) in the synovial fluid, which acts as mediators of tissue injury [4].

Currently there is no panacea for RA, but contemporary treatments aim to reduce inflammation, relieve pain (NSAID, corticosteroid) or alter the course of the disease such as disease-modifying antirheumatic drugs (DMARDs) or biologic therapies [5, 6]. Methotrexate (MTX) which is an antimetabolite and a DMARD is generally recognized as the first-line treatment for RA. It is designed in a way that synovitis and systemic inflammation are addressed effectively. However, that efficacy is hindered by the adverse effects it generates on the liver, rendering compliance to the treatment negligible. The toxic effects of MTX are suggested to be due to the accumulation of methotrexate polyglutamates in the liver resulting in the depletion of hepatic folate stores and inhibiting purine and pyrimidine precursor synthesis. Prolonged use of MTX renders the body defenseless against the sustained production of toxic free radical by overwhelming and eventually depleting the antioxidant defense system and in the process aiding in the initiation and progression of hepatotoxicity [6, 7]. Consequently, complementary and alternative natural agents are nevertheless desired and strongly recommended.

Of these natural agents, Indole-3-carbinol (I3C) is of interest. It is a naturally occurring compound found in cruciferous vegetables of the Brassica genus (all species of cabbage plants, black radish, garden radish, and mustard) [8, 9]. Several researches have confirmed that I3C has the following therapeutic actions: cancer chemopreventive [9–15], anti-inflammatory [9, 13–16] and anti-oxidant [16, 17]. I3C is usually given in diet or administered as a supplement considering the active constituent is formed under acidic conditions in the stomach. [11–13]. I3C acts as a potent anti-inflammatory agent by suppressing immune cells infiltration and pro-inflammatory cytokine production such as IL-1β, IL-6 and TNF-α in rodent models of inflammatory diseases [8]. It is one of several vegetable components that might protect against cancer. Considerable evidence shows that I3C inhibits experimentally induced carcinogenesis at different sites in the colon, lung, skin, liver, cervix and mammary gland in mouse and rat models [9, 13]. Furthermore, pre-treatment with I3C can attenuate experimentally- induced oxidative stress [8].

Although I3C possess many potential therapeutic activities, it was never tested on experimental arthritic models or used in conjugation with MTX to examine the therapeutic benefit of such combination. Adjuvant-induced Arthritis (AIA) is a well known experimental model of rheumatoid arthritis [18, 19]. The present study was designed to examine the therapeutic properties of I3C in AIA model on 1) the aberrant inflammation initiated in AIA rat model by testing its anti-inflammatory and anti-arthritic properties in compassion with MTX and 2) on the hepatotoxicity generated by the systemic consequences of arthritis and MTX treatment by assessing its anti-oxidant and hepatoprotective properties.

## Methods

### Animals

Experiments were performed on male Sprague Dawley rats weighing 180–220 g (Beirut Arab University, Lebanon). The rats were housed in constant conditions at 22 ± 2 °C under a 12 h light/ 12 h dark cycle with free access to standard pellet diet and water ad libitum.

### Experimental design

Male Sprague Dawley rats were injected subcutaneously with 0.1 ml CFA (Complete Freund’s adjuvant- heat killed Mycobacterium tuberculosis suspended in paraffin oil and mannide monooleate 1 mg/ml; InvivoGen, USA) into the footpad of the left hind paw. A booster subcutaneous injection of 0.1 ml was given into the tail on the same day and on the following day. Low concentrations of CFA were used to minimize animal morbidity and mortality. Forty-eight rats (*n* = 8) were divided into six groups. The groups used are as follows: a) a normal group injected subcutaneously with saline into the left footpad, b) a normal group injected subcutaneously with saline into the left footpad which received the vehicle used to dissolve I3C (10% DMSO, 10% Tween 80, 80% sucrose), c) a group injected with CFA that received no treatment, d) a group injected with CFA that received MTX (2 mg/kg/week for 6 weeks intraperitoneally), e) a group injected with CFA that received I3C (100 mg/kg/day for 3 weeks by diet) f) group injected with CFA that received both MTX (2 mg/kg/week for 6 weeks intraperitoneally) and I3C (100 mg/kg/day for 3 weeks by diet). All treatments began once the induction of arthritis was done. I3C was given for 3 weeks and not 6 weeks to examine the prolonged hepatoprotective effect of I3C against the continued weekly injections of MTX. The rats were gently restrained by using a decapicone which allowed for the efficient delivery of CFA which reduced the animals suffering and discomfort. Animals were sacrificed using anesthesia (sodium pentobarbital (50 mg/kg, IP)) followed by cervical dislocation. Total of 8 rats were used per all groups where half of the rats (*n* = 4) were sacrificed at day 23 to assess inflammatory and hepatoprotective markers. However, blood was withdrawn from the remaining 4 rats to asses for these rats the soluble inflammatory and liver enzyme markers. The remaining 4 rats were sacrificed on day 44 to assess hepatoprotective and oxidative stress markers. Animal welfare and experimental procedures were approved and carried out in accordance with the guidelines proposed by the Institutional Review Board (IRB) (2015A-0013-S-M-89) Beirut Arab University, Lebanon.

## Inflammatory markers

### Clinical evaluation

Swelling and inflammation in CFA injected hind paw was examined and monitored once per week to follow the course of the disease. The severity of arthritis was evaluated by using a macroscopic scoring system ranging from 0 to 4 to evaluate inflammation, erythema and deformity; 0 = no inflammation or erythema, 1 = mild, but definite redness and swelling of the ankle or wrist, or apparent redness and swelling limited to individual digits, 2 = moderate inflammation and erythema, 3 = severe inflammation and erythema, 4 = maximally inflamed limb with ankyloses or inability to bend the ankle or wrist [20]. The body weight of rats was monitored once weekly.

### Erythrocyte sedimentation rate (ESR) determination

**Fig. 1:**
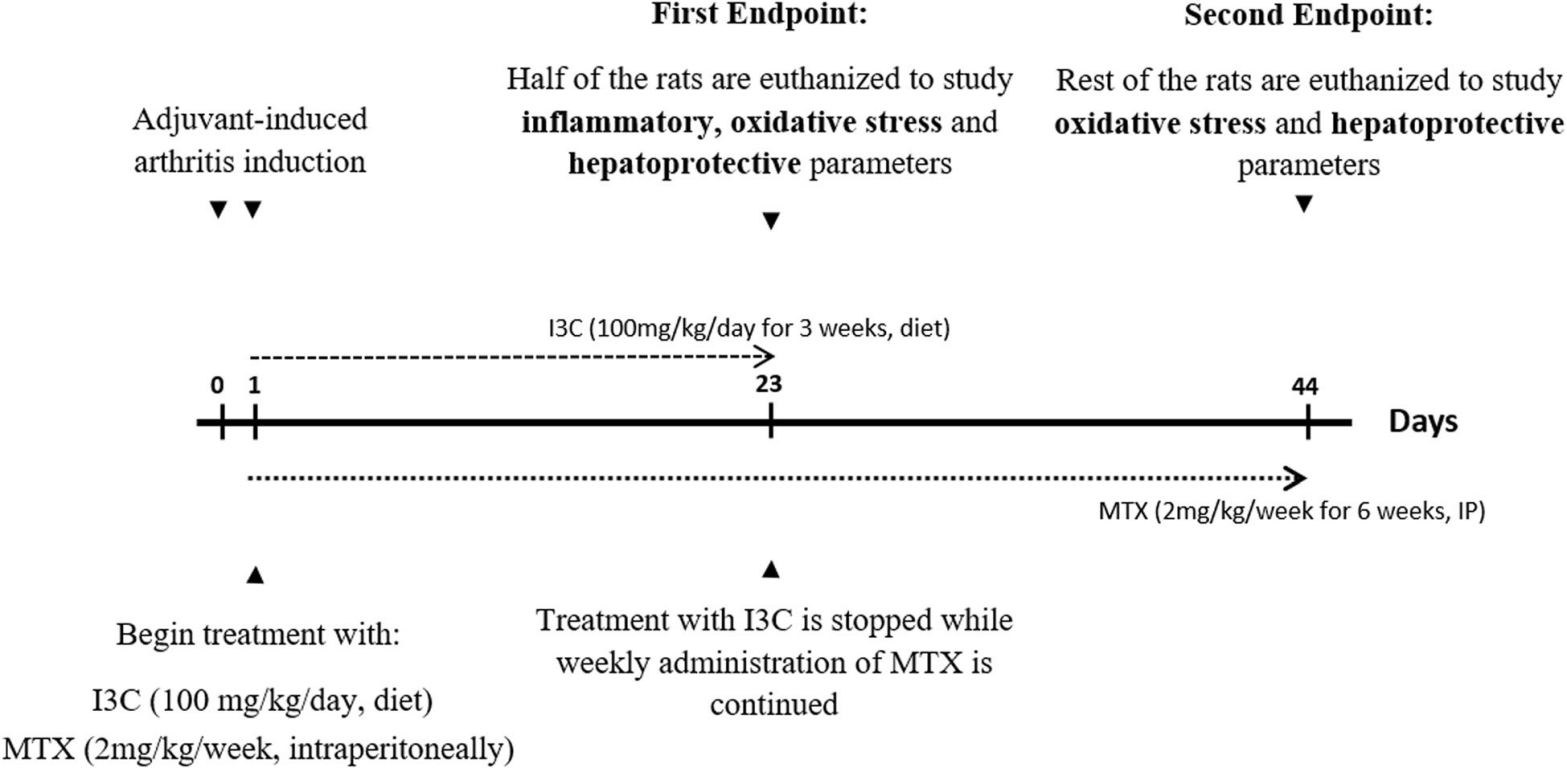
Experimental design: Induction of arthritic experimental model was accomplished on day 0 and 1. Treatment with I3C began on day 2 and lasted for 21 days only. Cessation of the treatment with I3C done to examine the prolonged hepatoprotective effects of I3C against the continued weekly injections of MTX. Treatment with MTX was administered weekly up to 6 weeks. Inflammatory markers were investigated only on the first endpoint (day 23) considering the inflammation that was induced on days 0 and 1 would resolve beyond the third week

Half of the animals were sacrificed using anesthesia followed by cervical dislocation on day 23 and whole blood was withdrawn from all the rats (Fig. 1). Erythrocyte sedimentation rate was determined by the Westergren method. The tubes were mounted in a vertical position and ESR was read 1 h later as mm of clear plasma [21].

### Serum TNF-α and IL-6 levels

The levels of the serum cytokines were determined by sandwich ELISA (Research and Development (R&D) Systems, Minneapolis) according to the manufacturers manual on day 23.

### Spleen index

The spleen was excised and weighed. The index of spleen was expressed as the ratio of spleen wet weight versus bodyweight (mg/g) on days 23 [22].

### Histological examination of the tibiotarsal joint

Left hind paws were excised where most of the skin and the digits were removed. Specimens were fixed in 10% Neutral Buffered Formalin and decalcified in a demineralizing solution containing 10% formic acid and 8% hydrochloric acid for 2 weeks. The ankle joints were transected in the longitudinal plane (sagittal) into two approximately equal halves (right and left) using the tibia as a guide and were then processed prior to staining [23]. The joints were stained with H&E, Masson’s Trichrome and Toluidine blue O staining. Pathological changes were scored (0–3) by a pathologist who was blinded to the treatment received. H&E stain was done to grade the histological changes occurring: synovial hyperplasia, cellular infiltration and pannus formation [24]. Toluidine Blue O stain was employed to assess the proteoglycan content in the non-calcified area of the cartilage. Loss of proteoglycan content is considered a sign of injury in the cellular component of the cartilage [25]. Masson’s Trichrome stain was however used to assess the presence of calcified cartilage. The presence of calcified cartilage in the superficial areas of the cartilage is considered a sign of a cartilage repair mechanism [24].

## Liver and oxidative stress markers

### Measurement of liver function enzymes

Liver dysfunction was evaluated by measuring serum levels of Alanine aminotransferase **(**ALT) and Aspartate aminotransferase (AST) using spectrophotometric kits (Analyticon® Biotechnologies AG. and Spinreact, S.A./S.A.U Ctra. Santa Coloma, respectively) on days 23 and 44.

### Preparation of liver tissue homogenate and measurement of total protein content

Part of the liver was removed, frozen in liquid nitrogen, and stored at− 80 °C for determination of total protein quantification, lipid peroxides malondialdehyde (MDA), catalase (CAT), superoxide dismutase (SOD) activities, and reduced glutathione (GSH) content on days 23 and 44. The total protein content was determined by Bradford’s method [26].

### Determination of lipid peroxidation levels (MDA)

MDA levels were determined in total liver homogenate spectrophotometrically at 523 nm as thiobarbituric acid-reactive substances (TBARS) [27]. The MDA content was calculated as TBARS and expressed in terms of nmol/mg of protein.

### Determination of glutathione (GSH) level

GSH was determined in total liver homogenate as described by Rahman and Biswas [28] by using a spectrophotometric assay which involves oxidation of GSH by the sulfhydryl reagent 5,5′-dithio-bis (2-nitrobenzoic acid) (DTNB) to form the yellow derivative 5′-thio-2-nitrobenzoic acid (TNB) at 405 nm. GSH content was calculated as nM per mg of protein.

### Determination of superoxide dismutase (SOD) activity

SOD was determined in liver homogenate as described by Beauchamp and Fridovich [29]. SOD activity was assayed by its ability to inhibit photochemical reduction of NBT at 560 nm. SOD was expressed as units per mg of total protein.

### Determination of catalase (CAT) activity

CAT was measured in liver homogenate according to Weydert and Cullen [30]. CAT activity was measured by a spectrophotometric procedure measuring peroxide removal. CAT was expressed as mmole per minute per mg of protein.

### Liver histology

Part of the liver taken was placed in 10% neutral buffered formalin and processed to perform histopathological assay. Paraffin sections were subjected to H&E staining and graded by a pathologist blinded to the treatment as follows: no observed changes; + mild changes; ++ moderate changes; +++ severe changes for both necrosis and inflammatory infiltration (lobular and portal).

### Statistical analysis

The obtained data was presented as mean ± standard deviation, difference between the groups was statistically determined by analysis of variance followed by posttest of Tukey for one-way analysis of variance and with Bonferroni adjustment for two-way analysis of variance, of significance set at *P* < 0.05. All statistical analyses were performed using GraphPad Prism 6.0 (GraphPad Software, San Diego, CA).

## Results

### Effect of I3C on the clinical parameters

**Fig. 2:**
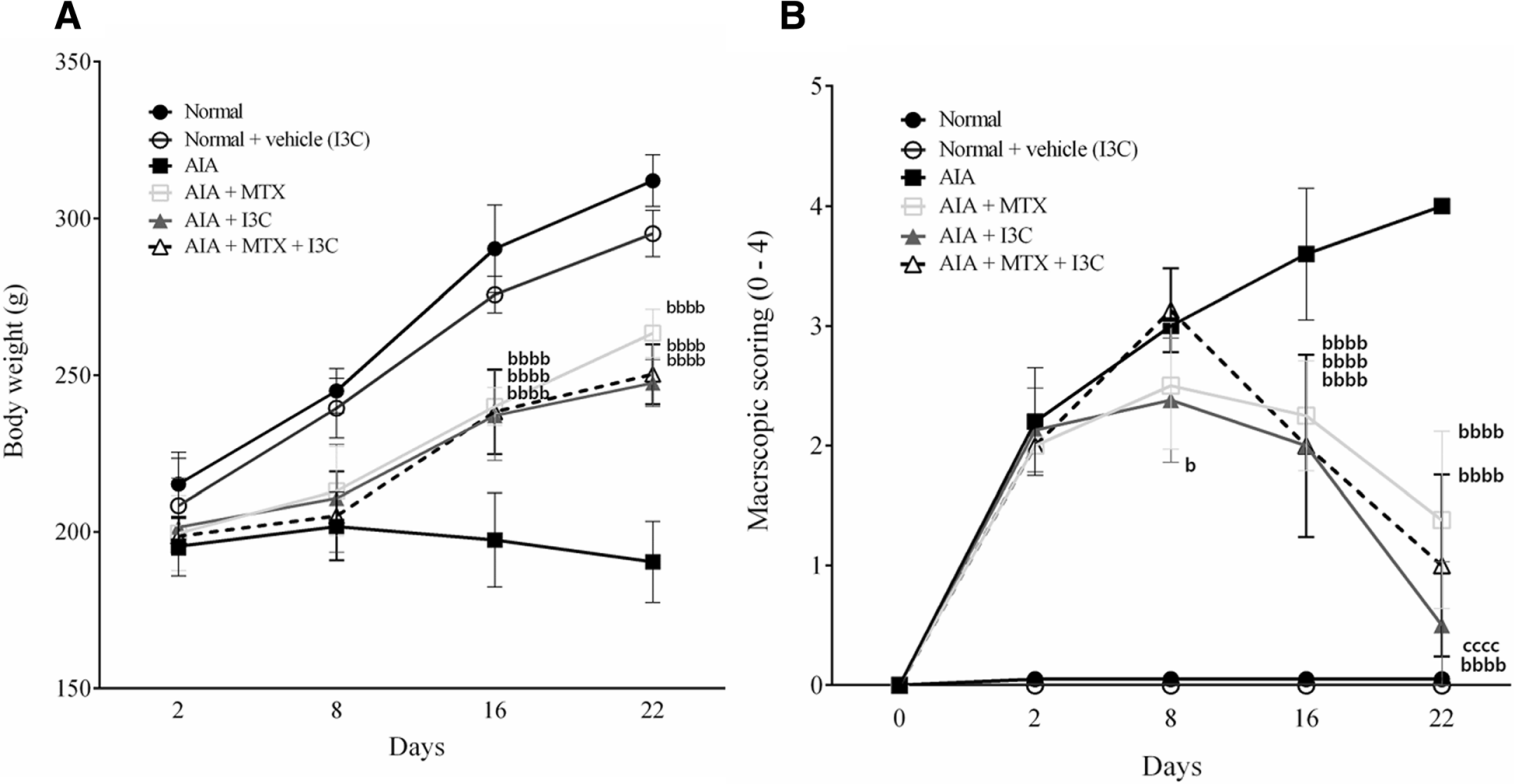
Assessing the effect of I3C by clinical evaluation. **a** Mean change in body weight of rats monitored once per week (*n* = 8). **b** Mean change in macroscopic score (0–4) of rats monitored once per week (*n* = 8). ^bbbb^*p* < 0.0001 compared with arthritic non-treated model group (AIA), ^cccc^*p* < 0.0001 compared with the positive control group (AIA + MTX)

Rats were evaluated once per week for clinical signs of arthritis by monitoring the body weight and inflammation affecting the injected paw. The body weight of rats in the two normal groups increased steadily from day 2 to 22 and these rats didn’t exhibit any inflammation or erythema as expected. However, the body weight of arthritic non-treated model group rats declined from day 2 to 22 compared to normal rats and these rats showed a gradual increase in the inflammation and erythema which were evident as increased macroscopic score with a peak of score 4 on day 22. On the other hand, arthritic rats treated with MTX, I3C, and the combination treatment demonstrated a marked increase in body weight compared to arthritic non-treated model group rats from day 2 to 22. Starting from day 16, arthritic rats treated with MTX, I3C and the combination treatment experienced alleviation from the inflammation affecting the paw where eventually on day 22 the inflammation became minimal. Rats treated with I3C experienced the greatest relief from inflammation and erythema which were evident as decreased macroscopic score (Fig. 2a, b).

### Effect of I3C on the soluble inflammatory mediators

**Fig. 3:**
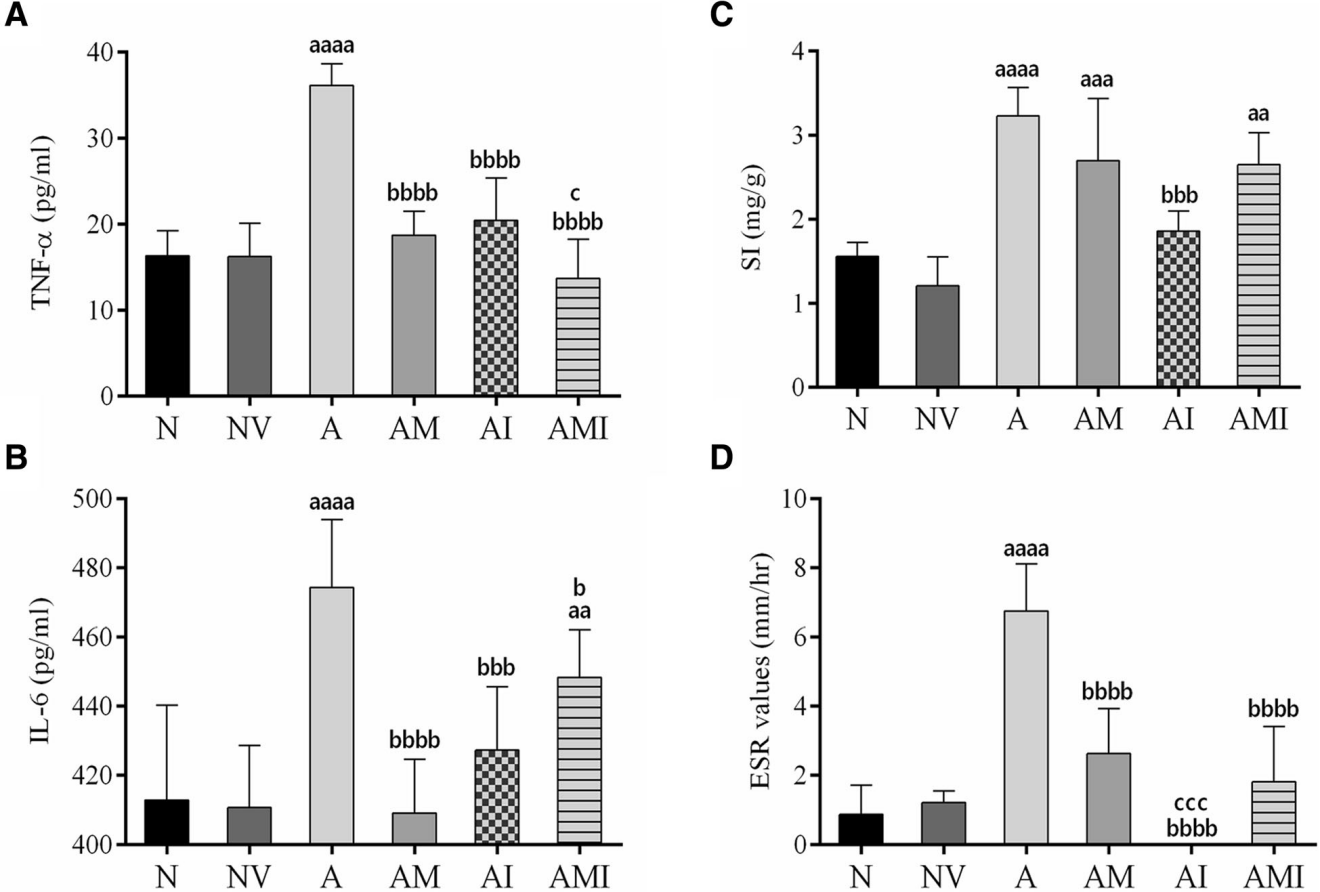
Assessing effect of I3C on inflammatory markers on day 23 (*n* = 8). **a** Mean TNF-α values (pg/ml) in serum of rats. **b** Mean IL-6 values (pg/ml) in serum of rats. **c** Mean spleen index values (SI) (mg/g) of rats. **d** Mean erythrocyte sedimentation rate values (ESR) (mm/hr) of rats. ^aa^*p* < 0.01 ^aaa^*p* < 0.001 ^aaaa^*p* < 0.0001 compared with the normal group receiving the vehicle (there was no statistical significant difference between the two normal groups), ^b^*p* < 0.05 ^bb^*p* < 0.01 ^bbb^*p* < 0.001 ^bbbb^*p* < 0.0001 compared with arthritic non-treated model group (A), ^c^*p* < 0.05 ^ccc^*p* < 0.001 compared with the positive control group (AM). N: Normal, NV: Normal + vehicle for I3C, A: AIA, AM: AIA + MTX, AI: AIA + I3C, AMI: AIA + MTX + I3C

There was no significant difference in serum TNF-α and IL-6 mean values between normal rats receiving the vehicle and those that didn’t (*P* > 0.05). Induction of the AIA model, however, lead to more than 2-fold increase in TNF-α and IL-6 values (*P* > 0.0001) when compared to normal rats. All treatments given significantly decreased TNF- α compared to arthritic non-treated model group rats (*P* < 0.0001) with no statistical difference when compared to normal groups (P > 0.05). The combination treatment provided further significant reduction when compared to the positive control group (*P* = 0.03) (Fig. 3a). IL-6 values decreased significantly when arthritic rats were treated with MTX (*P* < 0.0001), I3C (*P* = 0.0006), and the combination (*P* = 0.014). The combination treatment didn’t provide any further decrease (Fig. 3b).

### Effect of I3C on inflammatory markers and circulation of inflammatory cells

Arthritic non-treated model group exhibited an increased ESR value when compared to normal rats (*P* < 0.0001). However, arthritic groups treated with MTX, I3C and the combination showed more than 2 fold decrease when compared to arthritic non-treated model group,(*P* < 0.0001). There was no statistical significant difference between treated groups and normal groups (*P* > 0.05) (Fig. 3c). Spleen index (SI) acts an indicator for the status of inflammatory cell circulation where increased SI signifies increased circulation and recruitment of inflammatory cells. Induction of arthritis led to a significant increase in SI when compared to normal rats (*P* < 0.0001). Only arthritic rats treated with I3C demonstrated a significant decrease in the SI value compared to arthritic non-treated model group (*P* = 0.0006) (Fig. 3d).

### Effect of I3C on arthritic-induced pathological changes in the tibiotarsal joint in the ankle

**Fig. 4:**
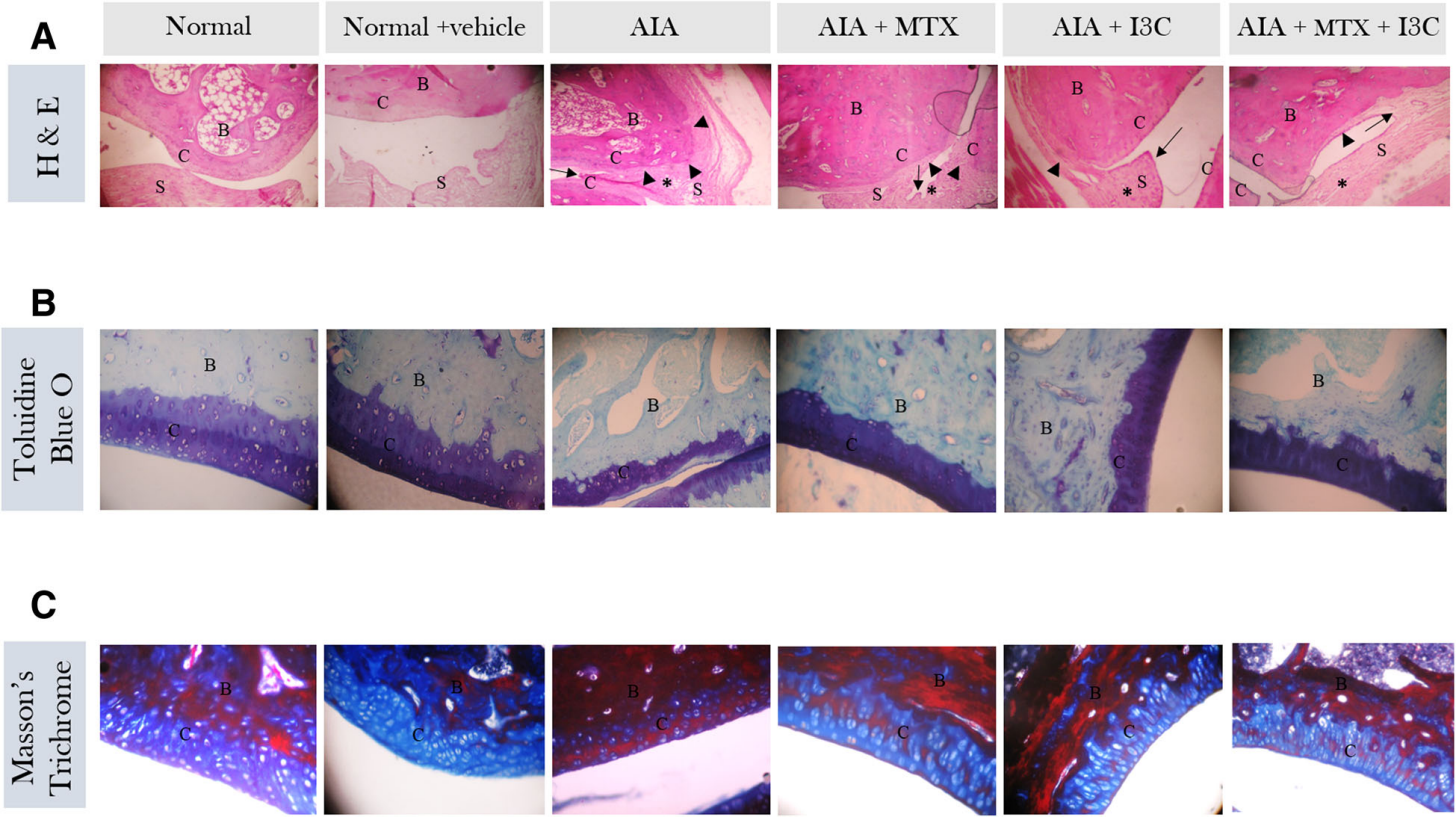
Studying effect of I3C on the tibiotarsal joint in the ankle at a histological level. **a **H&E staining was done to study cellular infiltration (*), synovial hyperplasia (**↑**), pannus formation (►). Arthritic non-treated model group reveals severe pathological changes while the treated groups reveal reduced pathological changes. **b, c** Toluidine Blue O was used to qualitatively assess the proteoglycan content in the cartilage while Masson’s Trichrome was used to qualitatively assess the presence of calcified cartilage. Toluidine Blue staining of arthritic non-treated model group reveal the presence of locally extensive areas of superficial matrix pallor accompanied by degeneration of chondrocytes while Masson’s Trichrome stain reveal substantial amounts of red color staining in the non-calcified cartilage region (superficial layer of cartilage) signifying the heavy presence of calcified cartilage. All treated groups reveal less damage exerted on the cartilage. B: bone, C: cartilage, S: synovium. Photomicrographs (A) at × 40; (B, C) at × 200

H&E staining of the tibiotarsal joint histology showed remarkable difference among the groups. The two normal groups revealed normal characteristics of the tibiotarsal joint in the ankle with no inflammation affecting the joints with a preserved synovium (grade 0). There was no cellular infiltration radiating from the synovium towards the capsule (grade 0), with no evidence of pannus formation growing or invading the cartilage and the bone (grade 0). However, the tibiotarsal joint of arthritic non-treated model group revealed severe synovial hyperplasia characterized by effacement of the joint space and adjacent cartilage (grade 3). There were extensive cellular infiltrates invading the capsule with the inflammation extending towards the skin (grade 3). In addition, invasive granulation tissue formation (pannus formation) was quite evident and extensive (grade 3). Arthritic groups treated with MTX diminished the pathological changes affecting the joint. Synovium hyperplasia was decreased to two - four layers of synoviocytes (grade 2). Cellular infiltration was still severe but with no aggregate formation, and the pannus formation was moderate (grade 2). Arthritic rats treated with I3C and the combination demonstrated even further significant reduction in these pathological changes when compared to the positive control group – MTX. The cellular infiltration was almost minimal with only few focal infiltrates and with mild pannus formation (grade 1). However, the synovial hyperplasia was similar to that exhibited by MTX treatment (grade 2) (Fig. 4a). Toluidine blue O staining revealed locally extensive areas of superficial matrix pallor accompanied by degeneration of chondrocytes in arthritic non-treated model group rats. All treated groups didn’t show any signs of proteoglycan loss similarly as the normal rats (Fig. 4b). Masson’s Trichrome staining revealed the presence of intact cartilage with blue staining only in normal rats. However, arthritic non-treated model group rats showed the presence of substantial amounts of red color staining in the cartilage, signifying the heavy presence of calcified cartilage. Arthritic groups treated with MTX or the combination treatment exhibited decreased damage exerted on the cartilage with the presence of moderate amounts of calcified cartilage. Arthritic groups treated with I3C alone demonstrated even further relief, with only mild calcified cartilage present (Fig. 4c).

### Effect of I3C on liver function enzymes

**Fig. 5:**
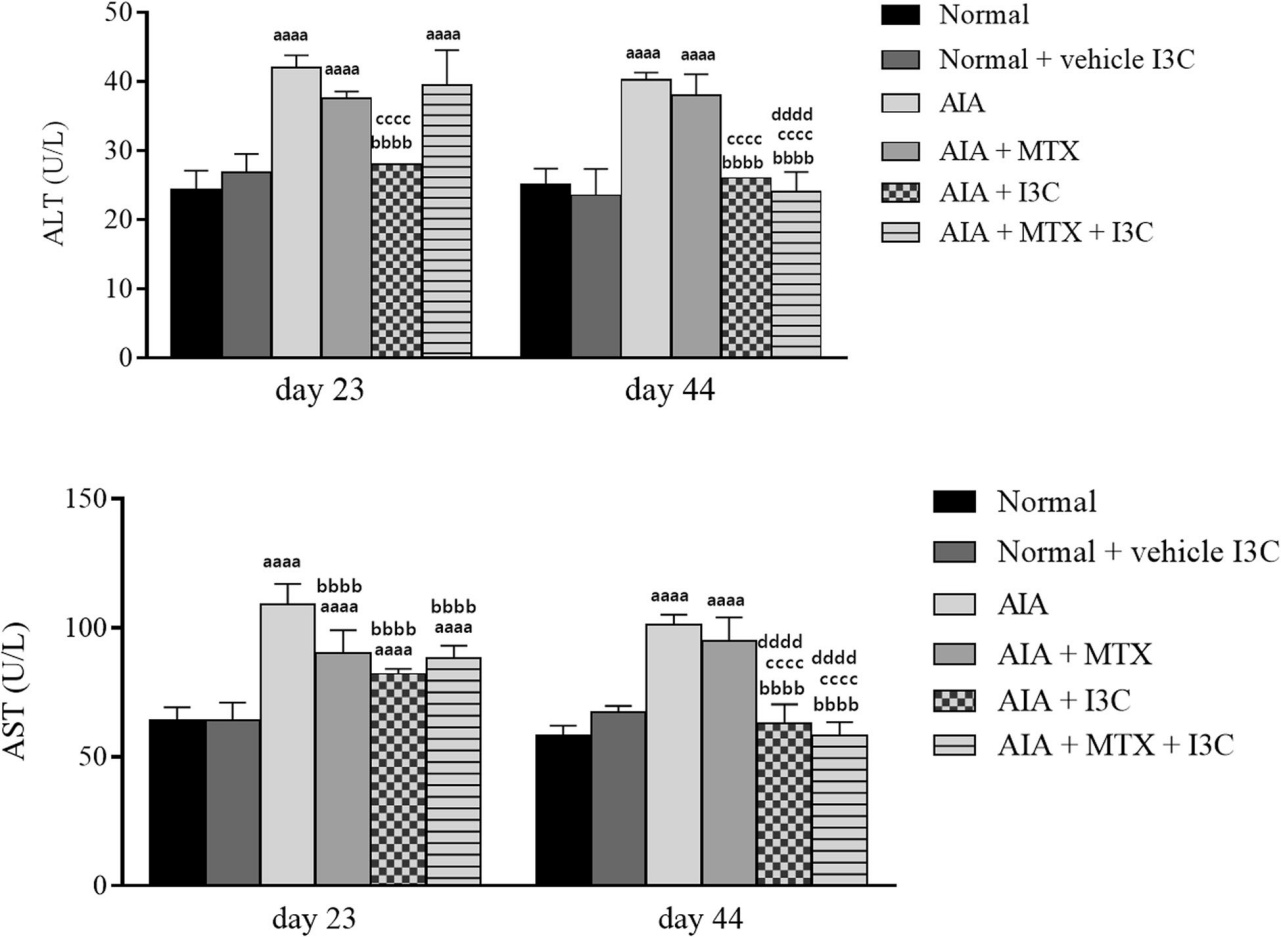
Assessing effect of I3C on liver function enzymes on day 23 and day 44 (*n* = 4). **a** Mean ALT values (U/L) in serum of rats. **b,** Mean AST values (U/L) in serum of rats. ^aaaa^*p* < 0.0001 compared with the normal group receiving the vehicle (there was no statistical significant difference between the two normal groups), ^bbbb^*p* < 0.0001 compared with arthritic non-treated model group (AIA), ^cccc^*p* < 0.0001 compared with the positive control group (AIA + MTX), ^dddd^*p* < 0.0001 compared with day 23

In order to assess the prolonged hepatoprotective effects of I3C against arthritis or MTX-induced liver injury, liver function enzymes (ALT and AST which are indicators of liver injury) were measured on days 23 and 44. Induction of arthritis led to elevations in ALT and AST on day 23 compared to normal rats (*P* < 0.0001) which remained elevated on day 44. Groups treated with MTX exhibited high ALT and AST on both days 23 and 44. Group treated with I3C showed decreased ALT and AST levels which was evident on day 23 (*P* < 0.0001). However, the combination treatment, exhibited ALT and AST values similar to the positive control group - MTX on day 23 (*P* > 0.05) which decreased significantly by 1.5 fold on day 44 (P < 0.0001) (Fig. 5a, b).

### Effect of I3C on arthritis and MTX-induced liver injury at a histological level

Liver toxicity was further evaluated by histopathological assessment of liver tissue in different groups. Both normal groups showed normal histological structure of the central vein with normal surrounding hepatocytes. Both groups revealed normal characteristics of the liver with no inflammation affecting portal or lobular tract (no observed changes) nor cellular infiltration. However, the liver of arthritic non-treated model group revealed moderate changes characterized by portal inflammation at day 23 and day 44 (++). However, more extensive cellular infiltration at day 44 was revealed (+++).

**Fig. 6:**
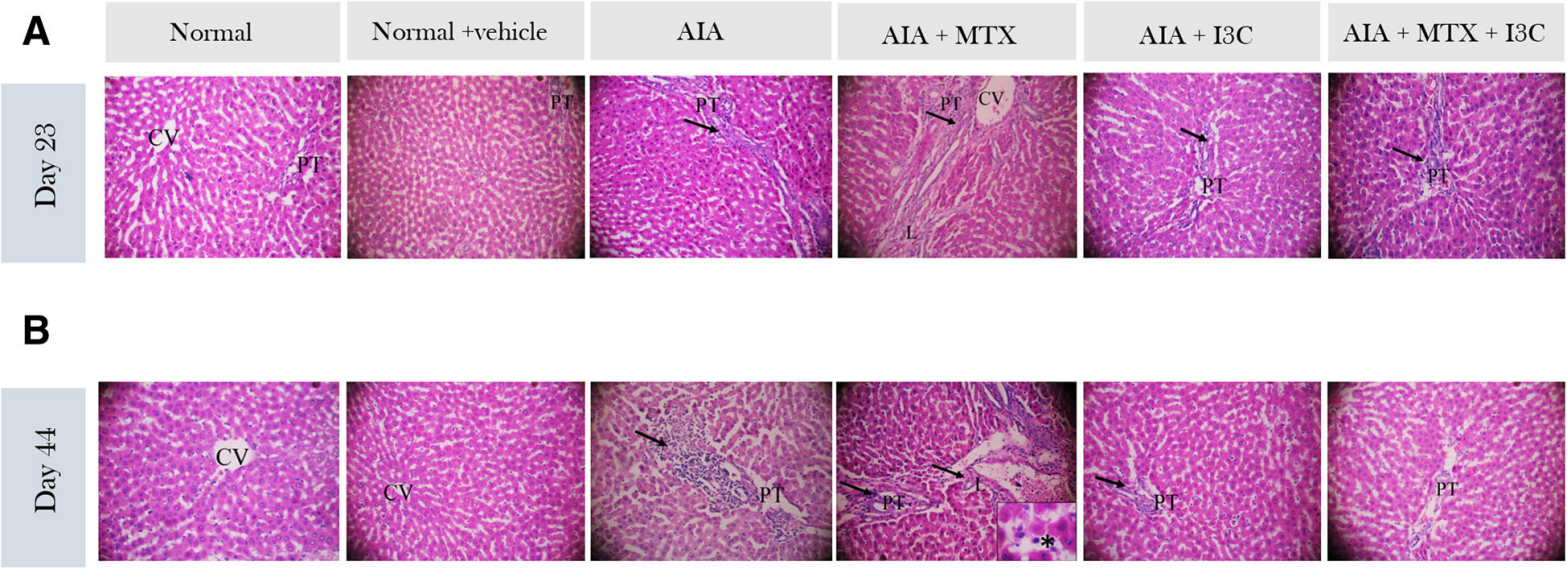
Studying effect of I3C on liver at a histological level on day 23 (**a**) and day 44 (**b**). H & E staining was employed to grade portal and lobular inflammation (→) and necrosis (*). Liver section of both normal groups show normal central vein (CV) and surrounding hepatocytes. Arthritic-non treated model group show inflammation in the portal tract. Treatment with methotrexate resulted in infiltration of cells in portal and lobular tract with mild congestion and necrosis (*) at day 44 (× 400). Treatment with I3C improved the architecture showing only mild portal inflammation. Liver section of arthritic group concurrently treated with MTX and I3C show portal inflammation with diffuse inflammatory cells (arrow). PT: portal tract, L: lobular, CV: central vein. Magnification is × 100 unless mentioned otherwise

MTX administration induced liver injury in the form of moderate portal inflammation (++) with moderate lobular inflammation accompanied with mild congestion in the portal tract at day 23. Extensive cellular infiltration invaded the liver with severe portal and lobular inflammation (+++) where necrosis was quite evident and prominent at day 44. Treatment with I3C alone revealed improvements in the liver histology when compared to arthritic non -reated group where only mild portal inflammation was shown at day 44. I3C and the combination treatment were able to protect the liver from MTX injury where moderate portal inflammation was revealed at day 23. I3C and the combination treatment reduced the pathological changes affecting the liver with mild portal inflammation only (+) after 44 days accompanied with minimal cellular infiltration. No signs of necrosis were revealed (Fig. 6a, b).

## Effect of I3C on oxidative injury parameters measure in liver homogenate

**Fig. 7:**
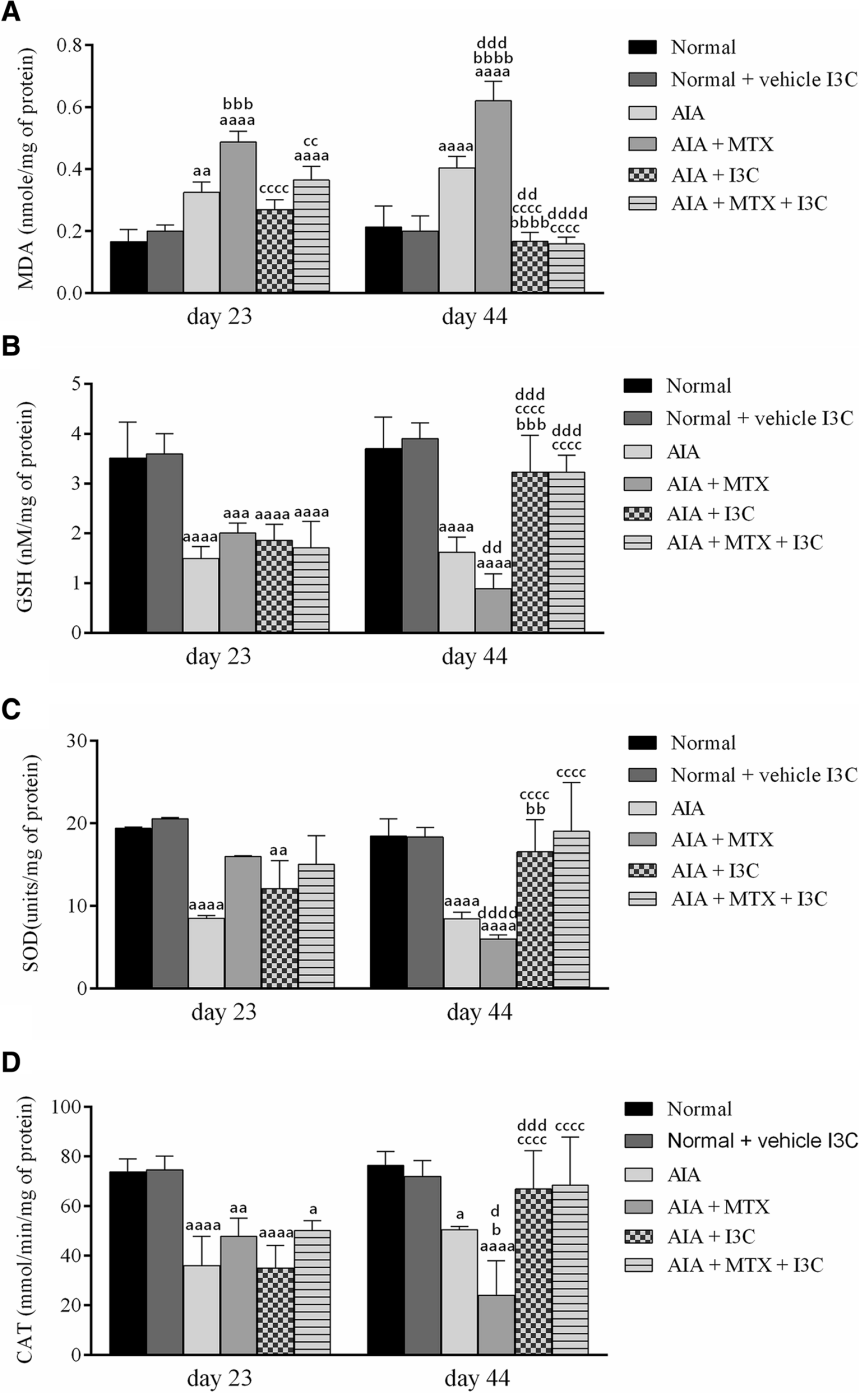
Assessing effect of I3C on oxidative stress parameters measured on day 23 and 44. **a** Mean malondialdehyde (MDA) levels (mmole/mg of protein) in liver homogenate of rats. **b** Mean Glutathione (GSH) levels (nM/mg of protein) in liver homogenate of rats. **c** Mean superoxide (SOD) activity (units/mg of protein) in liver homogenate of rats. **d** Mean catalase (CAT) activity (nmole/min/mg of protein) in liver homogenate of rats. ^a^*p* < 0.05 ^aa^*p* < 0.01 ^aaa^*p* < 0.001 ^aaaa^*p* < 0.0001 compared with the normal group receiving the vehicle (there was no statistical significant difference between the two normal groups), ^b^*p* < 0.05 ^bb^*p* < 0.01 ^bbb^*p* < 0.001 ^bbbb^*p* < 0.0001 compared with arthritic non-treated model group (AIA), ^cc^*p* < 0.01 ^cccc^*p* < 0.0001 compared with the positive control group (AIA + MTX), ^dd^*p* < 0.01 ^ddd^*p* < 0.001 ^dddd^*p* < 0.0001 compared with day 23

Induction of arthritis increased significantly the oxidative stress which was evident as an increase in MDA levels when compared to normal rats (*P* < 0.0001). However, weekly injections of MTX led to further significant increase in MDA levels compared to arthritic non-treated rat group on days 23 (*P* < 0.001) and 44 (*P* < 0.0001). Treatment with I3C decreased the MDA levels significantly on day 44 compared to arthritic non-treated model group rats and to the positive control group – MTX (*P* < 0.0001) with no significant difference when compared to normal rats (*P* > 0.05). The combination treatment reduced significantly MDA levels compared to the positive control group on day 23 and on day 44 by approximately 3-folds (*P* < 0.0001) (Fig. 7a).

Arthritic non-treated group rats exhibited compromised anti-oxidant defense system in the form of decreased CAT, SOD activities and GSH levels. Arthritic rats treated with MTX exhibited decreased anti-oxidant defense system (by more than 2 folds) with no significant statistical difference when compared to arthritic non-treated model group rats expect for catalase which was significantly lowered (*P* < 0.05). Rats treated with I3C and the combination treatment exhibited CAT, SOD activities and GSH levels similar to the normal rat groups on day 44 (P > 0.05) (Fig. 7b, c and d).

## Discussion

This study addressed the therapeutic benefits of I3C on the aberrant inflammation initiated by AIA model in comparison with MTX and its possible hepatoprotective and anti-oxidant effects against arthritis and the prolonged use of MTX. The main findings of this study were that daily administration of I3C for 21 days attenuates inflammation in a manner similar to MTX while alleviating the oxidative stress and hepatotoxicity generated by the prolonged use of MTX and by the systemic consequences of arthritis. Interestingly it even offers the possibility of prolonged protective benefits. Effects of the I3C on inflammation and oxidative stress have been previously addressed [14, 16, 31–34], whereas to our knowledge, this is the first report of investigating the therapeutic benefits of I3C when combined with MTX or on AIA rat model.

Consistent with findings from previous studies [7, 35, 36], our results clearly demonstrated that the injection of (1 mg/ml) CFA was associated with prominent arthritis in the injected paw. This was confirmed macroscopically and histologically at the tibiotarsal joint in the ankle using several types of specific stains. The H&E stain revealed the presence of classical destructive features of arthritis: proliferative synovitis accompanied with massive amounts of cellular infiltrates, aggregate formation and severe growth of destructive layers of granulation tissue. The latter was able to erode the cartilage which was proven by toluidine blue O and Masson’s Trichrome stain. Many cell types such as macrophages, lymphocytes, synovial cells and neutrophils aggregate into the articular cavity, releasing mediators and inflammatory cytokines [37, 38]. These released materials establish a complex niche that aggravate joint damage through pannus formation and cartilage erosion. The inflammation induced in CFA injected rats was evident as expected by the significant upregulation of serum cytokine levels (TNF- α and IL-6). This was accompanied by an increase in the circulation of inflammatory cells, which was confirmed by an increase in spleen weight and erythrocyte sedimentation rate. It has also been shown that cytokines released by inflamed synovial tissue can reach systemic circulation and act on other organs as proven in this study and by others [7, 21, 39, 40]. The systemic manifestations of arthritis were confirmed by a dramatic drop in the rats’ body weight and by impairing the liver. RA in humans is usually accompanied with oxidative stress and lipid peroxidation which is seen in animal models as well [7, 24]. Free radical induced hepatic damage due to impaired dynamic balance between prooxidant and antioxidant can initiate lipid peroxidation which was evident in this study by increased MDA levels. Induction of arthritis even aberrantly impairs the anti-oxidant defense system by almost depleting as evident here the vital line of defenses (GSH, SOD and CAT) against toxic free radicals [6]. This was even confirmed by increased ALT and AST levels in serum. The raised activity of these enzymes in serum is structurally related to the damage targeting the liver which leads to increased membrane permeability to ions resulting in their release into the circulation [21, 24]. Even the intensity of liver injury was revealed at histological level with increased inflammatory infiltration. These results were in accordance with the results of other studies that have investigated the action of CFA-induced arthritis in rats [7, 20, 24, 41–43].

The ameliorating effect of MTX therapy was evident as expected. Its well-known anti-arthritic and anti-inflammatory effects were proven on AIA model in this study and by others, too [6, 7, 21, 43]. The polyglutamate form of MTX (MTXGlu) is known to inhibit the enzyme 5-aminoimidazole-4-carboxamide ribonucleotide transformylase causing an increase in adenosine and cAMP levels that are responsible for the anti-arthritic and anti-inflammatory actions of MTX [21]. In this study, both MTX and I3C alleviated the severity of the disease by down regulating the pro-inflammatory cytokines (TNF-α, IL-6) and acting directly on the recruitment of inflammatory cells which was evident by a decreased ESR and spleen index. These cytokines play a pivotal role in recruiting immune cells, stimulating the release of matrix metalloproteinase and other proteinases to degrade cartilage, and upregulating the expression of pro-inflammatory mediators. Therefore, regulation of these cytokines in arthritic subjects is one of the approaches to treat arthritis [1, 44, 45]. This beneficial effect even extended to alleviating paw swelling and inflammation and inhibiting to a certain point the weight loss that was triggered by arthritis. The therapeutic effects of MTX and I3C were even evident microscopically at the histological level where they decreased the pathological changes (synovial hyperplasia, pannus formation, cellular infiltration and cartilage erosion) in the tibiotarsal joint in the ankle with better effects observed when I3C is used. The potent anti-inflammatory and anti-arthritic effects of I3C observed here corroborate with other studies. I3C has been shown to significantly decrease several inflammatory mediators including TNF-α and IL-6 in lipopolysaccharide-activated macrophages and in clonidine-induced neurotoxicity [8, 34].

Although proven quite effective, an adverse effect is known to limit the efficiency of MTX. Hepatotoxicity is known to occur in RA patients taking the therapeutic dose of MTX for a prolonged period of time as also evident in arthritic animal models [7, 21]. In this study, rats treated with MTX, exhibited increased lipid peroxidation (MDA) and decreased anti-oxidant defenses (CAT) compared to arthritic non-treated rats. This was even evident in the liver at a histological level with marked increase in the pathological scores. The toxic effects of MTX largely result from the depletion of hepatic folate stores (MTX is a folate antagonist) due to accumulation of methotrexate polyglutamates in the liver [46]. MTX is also known to increase the generation of toxic by-products such as reactive oxygen species (ROS) and inhibit the cofactors of several anti-oxidant enzymes ultimately decreasing the anti-oxidant defense mechanism known to protect the liver from damage [7, 21, 46]. This proves the need for alternative strategies to handle this deleterious adverse effect.

Although the concurrent treatment with MTX showed no synergistic activity regarding the inflammatory and arthritic markers assessed, I3C may have induced hepatoprotective and anti-oxidant effect against the well-known MTX toxicity and arthritis toxicity. I3C upregulated the anti-oxidant defenses (GSH, SOD and CAT) and in the process inhibited lipid peroxidation. This was even evident by decreased ALT and AST values. It should be noted that MTX treatment had no significant effect on ALT and AST levels in contrast to I3C treatment. I3C even attenuated the pathological changes affecting the liver at histological level that were induced by arthritis and MTX treatment (decreased cellular infiltration). These results are consistent with other studies that showed that I3C exhibits potent hepatoprotective effect against known hepatocarcinogenic agents such as diethylnitrosamine, 2-acetylaminofluorene and trabectedin) [47–49]. I3C even exerts potent anti-oxidant effects against hyperglycemia-mediated oxidative stress and clonidine-induced neurotoxicity [8, 50].

## Conclusion

Interestingly, in this study, I3C markedly attenuated the progression of the arthritic disease in manner similar to MTX but without impairing the liver or inducing oxidative stress as opposed to MTX. The potent therapeutic action of I3C largely revolves around its ability to significantly downregulate the release of inflammatory mediators and markers (TNF-α, IL-6 and ESR) from damaged tissues. I3C abrogated the oxidative stress and toxicity known to be induced by arthritis and MTX treatment. Considering the experimental model of arthritis used in this study shares several clinical and pathologic similarities to human RA [7, 35] then I3C could represent a novel therapeutic agent in the treatment of arthritis.

## Abbreviations

AIA: Adjuvant-Induced Arthritis
ALT: Alanine aminotransferase
AST: Aspartate aminotransferase
CAT: Catalase
CFA: Complete Freund’s adjuvant
DHF: Dihydrofolate
DMARD: Disease modifying antirheumatic drug
DMSO: Dimethyl sulfoxide
DTNB: 5,5′-dithio-bis-[2-nitrobenzoic acid]
ESR: Erythrocyte sedimentation rate
GSH: reduced glutathione
H&E: Hematoxylin and Eosin
I3C: Indole-3-Carbinol
IFN: Interferon
IL: Interleukin
IRB: Institutional Review Board
MDA: Malondialdehyde
MTX: methotrexate
MTX-Glu: Methotrexate polyglutamate
NO: nitric oxide
NSAID: Nonsteroidal anti-inflammatory drug
RA: Rheumatoid Arthritis
ROS: Reactive oxygen species
SD: Sprague-Dawley
SI: Spleen index
SOD: superoxide dismutase
TBA: Thiobarbituric acid
TBARS: Thiobarbituric acid reactive substances
TNB: 5-thio-2-nitrobenzoic acid
TNF-α: Tumor necrosis factor alpha
